# Supplementary material for: WordCommentsAnalyzer: A windows software tool for qualitative research
Source: F1000Res. 2018 Sep 4;7:536. Originally published 2018 May 3. [Version 2] doi: 10.12688/f1000research.14819.2 (PMC6137411; doi:10.12688/f1000research.14819.2)
Supplement: Supplementary file 3 [file f1000research-7-17614-s0002.tgz › b2a79496-4ada-47c4-9682-a99ce867836d.zip!/Eight Iranian Health Journals/Journal of Research in Rehabilitation Sciences _46.docx]

© Journal of Research in Rehabilitation Sciences . This work is licensed under CC BY-NC 4.0 (https://creativecommons.org/licenses/by-nc/4.0/).

Volume & Issue: دوره 6، شماره 2: 1389

Title: آیا ثبات مركزي تنه، اندام تحتاني زنان ورزشكار را در معرض آسيب‌‌دیدگی قرار می‌دهد؟

http://jrrs.mui.ac.ir/index.php/jrrs/article/view/125

چكيده نتایج تحقیقات نشان مي‌دهد که زنان ورزشكار نسبت به ورزشكاران مرد، هنگام فعاليت‌هاي ورزشي در اندام تحتاني بيشتر دچار آسيب‌دیدگی مي‌شوند. اين آسيب‌ها مي‌توانند با ثبات مركزي در ارتباط باشند. هدف از انجام اين پژوهش، بررسي ارتباط بین میزان ثبات مرکزی و وقوع آسیب‌های اندام تحتانی در زنان ورزشکار با استفاده از تست‌هاي عملكردي بود. آزمودنی‌های این تحقیق 50 نفر از زنان ورزشكار دانشگاهي در رشته‌هاي واليبال، بسكتبال و هندبال با ميانگين سن 4/1 ± 30/21 سال، قد 23/2 ± 06/163 سانتی‌متر و وزن 14/2 ± 92/59 کیلوگرم بودند. با توجه به سابقه آسيب در اندام تحتاني این گروه طی دو سال گذشته و محدوديت‌های موجود، تعدادی از آن‌ها به صورت هدفمند انتخاب شدند. در این تحقیق از فرم جمع‌آوري اطلاعـــات جهت ثبت آسيب‌هاي ورزشكاران استفاده شد. از تست‌هاي عملكردي برگرفته شده از تمرينات ثبات عمقي براي سنجش استقامت عضلات ثبات دهنده مركزي استفاده شد. همچنین تست استپ داون براي سنجش قدرت عملكردي عضلات كمري- لگني به کار گرفته شد. از روش آمار توصيفي همبستگي Pearson و آزمون z- Fisherبراي تجزيه و تحليل داده‌ها استفاده شد (05/0 = P). يافته‌‎هاي تحقيق نشان‌گر ارتباط معني‌دار منفي به نسبت بالايي بين ثبات مركزي و وقوع آسیب‌هاي اندام تحتانی در زنان ورزشکار بود (690/0- = r). همچنین بيشترين ارتباط بين قدرت عملكردي عضلات كمري- لگني و وقوع آسیب‌های اندام تحتانی ديده شد. با توجه به نتایج این پژوهش و همچنين در نظر گرفتن محدوديت تحقيــق در خصوص نحوه انتخاب آزمودني‌ها (هدفمند) نتیجه‌گیری می‌شود که ثبات مرکزی ضعیف به احتمال زیاد یکی از عوامل اثرگذار بر میزان آسیب‌دیدگی ورزشکاران زن، حداقل در آزمودني‌هاي مورد مطالعه باشد.

Title: بررسي مقايسه اي تاثير يك دوره تمرين در آب بر كيفيت زندگي مردان سالمند سالم

http://jrrs.mui.ac.ir/index.php/jrrs/article/view/126

چکیده مقدمه: جمعیت سالمندان در کشورهای در حال توسعه رو به افزایش بوده و همچنین سطح کیفیت زندگی این افراد به دلایل مختلف از جمله کمبود تحرک و فعالیت های جسمانی در حال کاهش است. تحقيق حاضر با هدف بررسی مقایسه ای تاثیر یک دوره تمرین در آب، بر کیفیت زندگی مردان سالمند انجام گرفت. مواد و روش ها: جهت انجام پژوهش 40 سالمند سالم بالای 60 سال به صورت تصادفی انتخاب و به همین روش به دو گروه تمرین (با ميانگين و انحراف استاندارد سني 5.3±66.2 سال، وزن 7.18 ± 62.5 کيلوگرم، قد 3.25 ± 166.5 سانتي‎متر و شاخص توده بدنی4.7±24.4) و گروه کنترل (با ميانگين و انحراف استاندارد سني 6.7±67.8 سال، وزن 4.6±63.5 کيلوگرم، قد 5.6 ± 164.3 سانتي‎متر و شاخص توده‎بدنی 4.3±23.3) تقسیم شدند. ابتدا همه شرکت‎کنندگان به سوالات پرسشنامه سنجش کیفیت زندگی لیپاد پاسخ دادند. سپس گروه تمرین به مدت 6 هفته، 3 جلسه در هفته و هر جلسه یک ساعت تمرینات خود را در آب اجرا کردند و گروه کنترل فقط کارهای معمول روزانه خود را انجام دادند. بعد از اتمام دوره تمرین مجددا هر دو گروه به سوالات پرسشنامه پاسخ دادند. پس از جمع آوری داده‎ها، نمرات پیش آزمون و پس آزمون با استفاده از آزمون های t وابسته و مستقل، کای- اسکوار و یو من ويتني مورد تجزیه و تحلیل قرار گرفت. نتایج: یافته ها حاکی از  تفاوت معنی‎دار بین میانگین نمرات پیش آزمون و پس آزمون گروه تمرین بود، چنین تفاوتی در نمرات پیش آزمون و پس آزمون گروه کنترل مشاهده نشد. همچنین نتایج نشان داد که بین میانگین نمرات پس آزمون دو گروه کنترل و تمرین، تفاوت معنی‎داری وجود دارد. بحث و نتیجه گیری: بر اساس یافته‎های این پژوهش، احتمالا به کارگیری تمرینات در آب می‎تواند عاملی تاثیرگذار در بهبود کیفیت زندگی سالمندان باشد. ‎

Title: بررسی و مقایسه تولید همخوانها در گفتار کودکان کاشت حلزون شده

http://jrrs.mui.ac.ir/index.php/jrrs/article/view/127

چكيده مقدمه : شنوايي مهمترين حس در رشد طبيعي گفتار و زبان است و كودكان ناشنوا به علت عدم دريافت مناسب محركهاي صوتي قادر به درك و توليد صحيح گفتار نبوده و وضوح كلامي آنها در سطح بسيار پاييني قرار دارد. انجام عمل كاشت حلزون با فراهم آوردن درونداد شنيداري مناسب، زمينه را جهت درك و توليد همخوانها فراهم مي آورد و انتظار مي رود در زمينه توليد و وضوح گفتار اين كودكان تغييرات زيادي را شاهد باشيم. بنابراين پژوهش حاضر با هدف بررسي و مقايسه توليد همخوانها در ارتباط با مدت زمان استفاده از سيستم كاشت حلزون انجام شد. مواد و روشها: نمونه هاي گفتاري 20 كودك كاشت حلزون شده در دو آزمون ناميدن تصوير و گفتار پيوسته از نظر تعداد خطاهاي توليدي مورد ارزيابي قرار گرفت و سپس براساس مدت استفاده از سيستم كاشت حلزون در 3 گروه مختلف آزمونهاي آماري جهت تحليل داده ها انجام شد. يافته ها: ميانگين خطاهاي توليدي در جايگاههاي مختلف همخوان در كلمه با مدتهاي مختلف استفاده از سيستم کاشت حلزون درآزمون ناميدن تصويربا 004/0 = Pدرسطح05/0 =α معني دار است. اما درآزمون گفتار پيوسته معني دار نمی باشد. نتيجه گيري: نتايج اين پژوهش نشان مي دهد كه با هر سال استفاده از سيستم كاشت حلزون خطاهاي توليدي در سطح تك كلمه به طور معني داري کاهش می یابد. اما جهت كاهش معني دار خطاهاي توليدي در گفتار پيوسته، زماني طولاني تر از 3 سال لازم است.

Title: بررسی تاثیر روش حرکت درمانی با محدودیت اجباری در بهبود کیفیت و عملکرد مهارتهای درون دستی کودکان مبتلا به فلج نیم تنه

http://jrrs.mui.ac.ir/index.php/jrrs/article/view/128

خلاصه مقدمه: از دیدگاه نظری مطرح می شود که محدودیت درمانی می تواند بر عدم استفاده یادگیری شده سمت مبتلا دربیماران همی پلژی غلبه کند و پلاستی سیتی مغزی را جهت ارائه الگوی کارآمدتر فعال سازد    Callautti&Baron,2003)). روش شناسی: این مطالعه یک مطالعه تک موردی) Single-Subject design) است که بر روی دو کودک با تشخیص خونریزی مغزی و فلج مغزی 9 و 10 ساله انجام شده است.اندام فوقانی سمت سالم در یک اسپلینت قابل برداشت  با جنس پلی پروپیلن برای 21 روز متوالی قرار داده شد.کودکان در یک برنامه درمانی فشرده در کلینیک و منزل به مدت 7 ساعت در روز شرکت کردند. ارزیابی های روزانه بر اساس کیفیت و عملکرد مهارتهای درون دستی  بر اساس 6 خرده آزمون در دست مبتلای کودکان انجام شد. تغییرات در 3 مرحله پیش از مداخله، مداخله و پس از مداخله (پیگیری) به کمک محاسباتC statistic مورد بررسی قرار گرفت. یافته ها و بحث: اگرچه وجه تمایز این مطالعه با مطالعات گذشته بررسی میزان تاثیر روش محدودیت درمانی روی مهارتهای درون دستی بر اساس تقسیم بندی جدید در سال 2009 بود، نتایج مطالعه حاضر نشان داد که این روش می تواند در بهبود کیفیت و عملکرد مهارتهای درون دستی کودکان همی پلژی خفیف موثر واقع شود (p<0.05; Zscore >1.68).

Title: مقایسه تاثیر اولتراتون واولتراسوند در سندرم کانال کارپ ایدیوپاتیک خفیف یا متوسط

http://jrrs.mui.ac.ir/index.php/jrrs/article/view/130

مقدمه: سندرم کانال کارپ یکی از شایعترین نوروپاتی های انسدادی است که درمان های متعددی (جراحی و غیر جراحی) برای آن پیشنهاد شده است. در فیزیوتراپی درمان های متنوعی برای آن پیشنهاد شده است که از جمله می توان استفاده از اسپلینت، نوروموبیلیزیشن، لیزر، اولتراسوند و اولتراتون را نام برد. هدف از این مطالعه مقایسه اثر تسکینی اولتراسوند و اولتراتون در درمان CTS (Carpal Tunnel Syndrome)  ایدیوپاتیک خفیف یا متوسط می باشد. (Trial Clinical)مواد و روشها: این مطالعه به شکل کارآزمائی بالینی بوده و روش نمونه گیری به صورت غیراحتمالی آسان بود.68 دست آسیب دیده مربوط به 59 بیمار، در دو گروه آزمایشی جداگانه  A اولتراتون(UT)و B اولتراسوند(US)  به طور تصادفی و بر اساس ترتیب مراجعه مورد مطالعه قرار گرفتند. شرایط ورود به مطالعه عبارت بود از درد و بی حسی در دست (بویژه در شست، انگشت اشاره و انگشت میانی)، بیدار شدن در شب، داشتن علائم برای مدت بیشتر از یک ماه، تست فالن مثبت، تست تینل مثبت، زمان تأخیری دیستال حرکتی بیشتر از 4.4 میلی ثانیه  (Onset)و  زمان تأخیری دیستال حسی بیشتر از 3.7 میلی ثانیه (Peak). در هر دو گروه UT و US، 10 جلسه درمان با استفاده از هات پک به مدت 15 دقیقه و اولتراتون یا اولتراسوند، به صورت روزانه انجام شد. .پارامترهای مورد بررسی در این مطالعه شامل درد و معیارهای الکترودیاگنوستیک بود. پارامتر درد قبل از شروع درمان و در آغاز جلسات سوم، پنجم، هفتم، نهم و چهار هفته پس از انجام فیزیوتراپی (جمعاً 6 بار) با استفاده از VAS و معیارهای الکترودیاگنوستیک صرفاً قبل و چهار هفته پس از فیزیوتراپی اندازه گیری شد. نتایج: میزان بهبودی یا کاهش درد در گروه درمانی اولتراتون بیشتر و سریعتر از گروه درمانی اولتراسوند دیده شد.هر چند این تغییرات اختلاف معنی دار آماری نشان ندادند (P<0.05).اثرات تسکینی این مدالیته ها در دوره ی Follow up ادامه پیدا نکرد و در ارزیابی نهایی در چهار هفته پس از درمان میزان درد افزایش یافته بود در هر دو گروه درمانی پارامترهای تأخیر حرکتی دیستال و تأخیر حسی دیستال چهار هفته پس از درمان هرچند کاهش یافته بود اما تغییرات با بررسی قبل از درمان از نظر آماری اختلاف معنی داری نداشت(P<0.84).تغییرات در سرعت هدایت الیاف حسی بیشتر از الیاف حرکتی بود و این تغییرات در گروه اولتراتون مشهودتر بود. در هر دو گروه آمپلی تود موتور و سنسوری در بررسی نهایی پس از درمان افزایش یافته بود اما این تغییرات نیز از لحاظ آماری معنی دار نبود(P<0.61) بحث: نتایج این تحقیق نشان داد در درمان بیماران مبتلا به  CTS  خفیف تا متوسط، اولتراتون از نظر تعداد جلسات مراجعه جهت کاهش درد می تواند تأثیر سریعتری داشته باشد. پیشنهاد می شود در درمان با مدالیتی های متداول فیزیوتراپی برای کنترل درد که موفقیت حاصل نمی شود از اولتراتون، اولتراسوند یا ترکیبی از آنها استفاده شود

Title: بررسي ميزان کارائي پروتزهاي اندام فوقاني در انجام فعاليت هاي مختلف درافراد آمپوته اندام فوقاني

http://jrrs.mui.ac.ir/index.php/jrrs/article/view/131

افراد قطع عضواندام فوقاني با کاهش چشمگيرتوانايي در انجام فعاليت هاي خود روبرو هستند براي جبران اين امر از انواع مختلف پروتز استفاده مي شود که مطابق با مکانيزم حرکتي ممکن است کازمتيک، مکانيکال و يا مايو الکتريک باشند. متاسفانه علي الرغم تلاشهاي فراوان در زمينه افزايش کارائي پروتزهاي اندام فوقاني ، ميزان موفقيت افراد دربه کاربردن پروتز پائين مي باشد‍‍؛ لذا در اين تحقيق سعي بر آن است تا ميزان کارائي پروتزها با توجه به انواع فعاليت مورد بررسي قرار گرفته، ارتباط آن با نوع پروتز، سمت قطع عضو و سطح قطع عضو تعيين گردد. روش مطالعه: تعداد 76 نفر از آمپوته هاي اندام فوقاني در استانهاي تهران، اصفهان و شيراز که به مراکز ارتوپدي فني مراجعه کرده بودند مورد بررسي قرار گرفتند. فاکتورهايي همچون ميزان توانائي افراد در انجام فعاليت هاي مختلف، سن فرد، طرف قطع عضو، سطح قطع عضو انتخاب گرديد. نتايج: نتايج اين تحقيق نشان داد که علي الرغم سيستم حرکتي به کار گرفته در پروتز؛ اکثر افراد از پروتز خود فقط در حد يک تکيه گاه استفاده مي کنند. ميزان توانايي آمپوته هاي اندام فوقاني در انجام فعاليت هاي مختلف همانند رانندگي كردن ونيازهاي شخصي از فعاليتهاي ديگربيشتر بود. گرچه انتظار ميرفت كه پروتزهاي هاي مکانيکال نيازهاي فرد را بهتر برآورده سازند، با اين حال در خيلي از سطوح قطع عضوي تفاوت ملموسي مشاهده نشد (p-(value = 0.1.   بحث و نتيجه گيري: از ميان فاکتورهاي مختلفي که مي تواند بر ميزان کارکرد پروتزهاي اندام فوقاني اثر گذار باشد، تاثير آموزش در استفاده از پروتز از اهميت بسزايي برخوردار است. پيشنهاد مي شود از تکنيک هاي جديد و مواد مناسبتر جهت بالا بردن کارائي پروتز ها در اين نوع از آمپوته ها استفاده گردد.

Title: بررسی اختلالات عضلانی اسکلتی بخش انتهایی اندام فوقانی کارگران صنعتی

http://jrrs.mui.ac.ir/index.php/jrrs/article/view/132

چکیده مقدمه: هدف این پژوهش بررسی اختلالات اسکلتی عضلانی بخش انتهایی اندام فوقانی در کارگران صنعتی بود. مواد و روش ها: جهت اجرای این پژوهش 50 نفر از بخش اداری و 160 کارگر صنعتی مشغول به پرس کاری، جوشکاری و کار با دستگاه دریل در کارخانه صنعتی سایپا انتخاب شدند. در این پژوهش از پرسشنامه نوردیک، روش مشاهده ای، روش مصاحبه ای و روش شاخص تنش جهت بررسی میزان خطرات پست های کاری مختلف استفاده شد. آزمون های تی زوجی و مستقل، آنالیز واریانس یک طرفه و آزمون تعقیبی توکی در برنامه  SPSS17 جهت تجزیه و تحلیل داده ها مورد استفاده قرار گرفتند( 05/0≥α). یافته ها: نتایج تحقیق نشان داد پست کاری جوش کاری، پرس کاری و کار با دستگاه دریل به ترتیب بالاترین شیوع اختلالات بخش دیستال اندام فوقانی را دارند. نتایج تحقیق نشان داد افراد درگیر در بخش های اداری دارای شرایط کاری ایمن با میانگین شاخص تنش 7/1، افراد درگیر در بخش جوشکاری دارای شغل با ریسک اندک با میانگین شاخص تنش 6/5، افراد درگیر در بخش پرس کاری و کار با دریل دارای نوع شغل مخاطره آمیز به ترتیب با میانگین شاخص تنش 2/7 و 1/8 بودند. نتیجه گیری: با توجه به نتایج تحقیق حاضر نتیجه گیری می شود افرادی که در معرض کارهای دارای ارتعاش فراوان قرار دارند، استعداد بیشتری جهت ابتلا به اختلالات اسکلتی- عضلانی قرار دارند.

Title: مقایسه تاثیر تمرینات تعادلی، ذهنی و ترکیبی بر تعادل مردان سالمند سالم

http://jrrs.mui.ac.ir/index.php/jrrs/article/view/133

چکیده مقدمه: از آن جائی که حفظ تعادل[1] یکی از شاخص های تعیین استقلال افراد سالمند به شمار می رود، بررسی و تشخیص عوامل موثر بر تغییرات تعادل بویژه اثرات انواع قراردادهای[2] تمرین ورزشی بر تعادل از موضوعات قابل بررسی است که مورد توجه محققان قرار گرفته است. هدف مطالعه حاضر بررسی مقایسه ای اثر تمرینات تعادلی در آب، تمرینات ذهنی و تمرینات ترکیبی (ذهنی و تعادلی در آب) بر تعادل مردان سالمند سالم بود. مواد و روش ها: تعداد 120 نفر از سالمندان، سن 14/14 ±07/65 سال، وزن 31/3±86/71 کیلوگرم و قد 57/3 ±2/173 سانتی متر، انتخاب و به صورت تصادفی در 4 گروه] گروه اول تمرینات تعادلی در آب (30 نفر)، گروه دوم تمرينات ذهني (30 نفر)، گروه سوم تمرین ترکیبی تعادلی در آب و تمرين ذهني (30 نفر)، گروه چهارم بدون تمرین یا کنترل (30 نفر)[ طبقه‎بندی شدند. براي برآورد تعادل آزمودني ها از آزمون Y[3] در سه جهت استفاده شد. سپس در مورد گروه تمرینی تعادلی در آب، تمرینات در آب با هدف افزایش تعادل آزمودنی‎ها به مدت شش هفته و هر هفته به مدت سه جلسه و هر جلسه به مدت یک ساعت انجام شد. در مورد گروه تمرینات ذهنی مدت تمرين ذهنی در هر جلسه 15 دقيقه و طول دوره مشابه گروه اول بود. در مورد گروه تمرین ترکیبی تعادلی در آب و تمرینات ذهنی، آزمودنی‎ها ترکیبی از تمرینات دو گروه قبل را انجام دادند. براي تجزیه و تحلیل آماری داده های خام از آزمون های آمار توصيفي، t همبسته، ANOVA و آزمون تعقيبي توكي (p≤0/05) استفاده شد. یافته ها: نتایح نشانگر افزایش فاصله دستیابی آزمودنی های سه گروه تمرینی پس از دوره تمرین بود. لیکن در مقايسه بين گروهها بر اساس مقادير دامنه اختلاف فاصله دستيابي قبل و بعد از اعمال تمرينات که اندازه دقيق تري از تأثير انواع تمرينات را بر تعادل فراهم مي کند، مشاهده مي شود که در هر سه جهت آزمون Y بيشترين افزايش فاصله دستيابي پس از اعمال دوره تمرين مربوط به گروه تمرينات تركيبي است. بحث و نتیجه گیری: نتایج تحقیق حاضر در توافق با یافته های قبلی نشانگر تاثیر معنی دار اجرای یک دوره تمرین ورزشی بر بهبود تعادل بود. دلالیل احتمالی افزایش تعادل در نتیجه تمرین شامل افزایش قدرت اندام تحتانی آزمودنی ها، تسهیل در هدایت عصبی عضلانی، افزایش سینرژی عضلانی، اعمال فشار بر سیستم عصبی عضلانی و مهار فرایند خودبخودی می باشد. با توجه به یافته های تحقیق حاضر می توان پیشنهاد کرد که در طراحی برنامه های آمادگی جسمانی ویژه سالمندان به تمرینات در آب و تمرینات ذهنی توجه خاص شود.

Title: بررسي ارتباط مؤلفه P100 در پتانسل برانگيخته بينايي با اختلال خواندن در بين كودكان دبستاني

http://jrrs.mui.ac.ir/index.php/jrrs/article/view/135

مقدمه: اختلال رشدي خواندن نوعي از نقص در خواندن و نوشتن است كه در آن فرد مبتلا داراي هوش طبيعي است و از مشكلات آموزشي و يا عاطفي رنج نمی برد با وجود عوارض رواني و پيامدهاي ناشي از عدم توانايي كافي در خواندن و نوشتن در زندگي فردي و اجتماعي مبتلايان، علت شناسي‌های آن به طور كامل مشخص نيست و روش تشخيص ي بيولوژيك براي آن پيدا نشده است. هدف از اين مطالعه مقايسه ميانگين دامنه وديرش موج P100 در دو گروه ديس لكسيك و غير ديس لكسيك و دو حالت مختلف محرك بينايي و استفاده از VEP جهت تشخيص ديس‌لكسيا بوده است . مواد و روش‌ها: پژوهش حاضر يك مطالعه تحليلی - مقطعی بود که 20 كودك ديس لكسيك و 20 كودك غير ديس لكسيك از پايه‌های دوم تا پنجم دبستان شهر اصفهان را مورد بررسي قرار داد كه به صورت تصادفي انتخاب شده بودند. تمام افراد در دو حالت مختلف تمايز محرك يبيناي تحت آزمون VEP قرار گرفتند. افراد ديس لكسيك به وسيله آزمون سياهه خواندن مورد شناسايي قرار گرفته بودند . يافته‌ها: كاهش تمايز سبب افزايش معنی‌دار ديرش موج P100 در تمام افراد شد يول ، مقايسه بين گروه معنی‌دار نبود. اما كاهش تمايز سبب كاهش ناچيز دامنه موج P100 در افراد غير ديس لكسيك و افزايش دامنه در افراد ديس لكسيك شد. تفاوت‌های بين فردي و بين گروهي دامنه موج P100 معنی‌دار نبود . نتيجه‌گيري: با توجه به آن كه تغيير تمايز محرك بينايي در آزمون VEP سبب تفاوت معني دار بين گروه‌ها نشد، روش مناسبی براي تشخيص ديس لكسيا نمي‌باشد. همچنين به دليل آن كه افراد ديس لكسيك در تمام شرايط برگزاري آزمون، عملكردي مشابه يا بهتر از غير ديس لكسيك‌ها داشتند، نظريه نقص مگنوسلولار در ديس لكسيك‌ها تأييد نمی‌شود.

Title: MSمقايسه تأثير تمرين درماني و بيوفيدبك روي بي اختياري ادرار بيماران مبتلا به

http://jrrs.mui.ac.ir/index.php/jrrs/article/view/136

چكيده عنوان: مقايسه تأثير تمرين درماني و بيوفيدبك روي بي اختياري ادرار افراد مبتلا به M.S . مقدمه: مولتيپل اسكلروزيس شايع ترين بيماري نورولوژي پيشرونده در افراد جوان بالغ مي باشد. اين بيماري باعث تخريب ميلين در آكسونهاي عصبي شده و در نتيجه ضايعات متفاوتي را به دنبال دارد كه يكي از اين مشكلات، اختلالات ادراري است. مشكلات ادراري، جنبه هاي مختلف زندگي روزانه ي بيمار را تحت تأثير قرار داده و يك تأثير منفي بر روي كيفيت زندگي اين بيماران به دنبال دارد. تمرين درماني و الكتروتراپي به عنوان دو روش موثر درماني در بهبود اختلالات مربوط به مشكلات ادراري بيماران MS قلمداد مي شوند كه نياز به تحقيقات گسترده تر در اين زمينه لازم به نظر مي رسد. مواد و روش ها: اين مطالعه از نوع آينده نگر، شبه تجربي،غيرتصادفي و يك سويه كور بوده كه در سال 87-1386 در كلينيك هاي فيزيوتراپي وابسته به دانشكده علوم توانبخشي اصفهان انجام شده است. در اين مطالعه 42 بيمار با داشتن شرايط ويژه، انتخاب و به صورت تصادفي به دو گروه 21 نفره تقسيم شدند كه طي 10 جلسه به صورت يك روز در ميان، يك گروه تحت درمان تمرينات سوييس بال و گروه ديگر تحت درمان بيوفيدبك قرار گرفتند. اطلاعات حاصل از انجام تمرينات، قبل از درمان، بعد از درمان و هنگام Follow  up يك ماهه جمع آوري گرديد.به منظور بررسي ميزان بي اختياري ادرار از پرسشنامه ICIQUISF و براي بررسي كيفيت زندگي از پرسشنامه SF-36 و براي بررسي تأثير بي اختياري ادرار روي انجام فعاليتهاي روزمره(ADL ) بيماران از پرسشنامه P.I.I.Q استفاده شد. جهت تجزيه و تحليل آماري نتايج به دست آمده از آزمون هاي موجود در نرم افزار13 SPSS استفاده شد.  نتايج:  پس از بررسي هاي آماري مشخص گرديد كه بين ميزان بي اختياري ادرار بيماران قبل از درمان و بعد از درمان هم در گروه سوييس‌بال و هم در گروه بيوفيدبك اختلاف معني داري وجود داشت (05/0< P). همچنين بين كيفيت زندگي بيماران هم از نظر فيزيكي و هم از نظر روحي ـ رواني، قبل از درمان و بعد از درمان، در هر دو گروه سوييس بال و بيوفيدبك، اختلاف معني داري وجود داشت (05/0< P). بحث: نتايج حاصل از اين مطالعه نشان داد كه هم تمرينات سوييس بال و هم بيوفيدبك باعث كاهش ميزان بي اختياري بيماران، بهبود كيفيت زندگي و انجام فعاليتهاي روزمره بيماران مي شود، ولي تمرينات سوييس بال در تداوم تأثير خود در كاهش ميزان بي اختياري ادرار و بهبود كيفيت زندگي بيماران از نظر فيزيكي نتايج بهتري را نسبت به بيوفيدبك از خود نشان داد. همچنين بيوفيدبك در زمينه انجام فعاليتهاي روزمره نتايج بهتري را نسبت به تمرين درماني از خود بر جا گذاشت.

Title: روي نقاط طب سوزني در بيماران مبتلا به سندرم درد مايوفاشيال در ناحيه گردن Ga. AL. AS تعيين اثرات درماني ليزر كم توان

http://jrrs.mui.ac.ir/index.php/jrrs/article/view/137

چكيده مقدمه: سندرم د رد مايوفاشيال (MPS ) ازشايعترين علل درگيري عضلات اسكلتال با منشاء تريگر پوينت  در يك يا چند عضله و يا بافت همبند است كه ميزان شيوع آنرا در مطالعات مختلف از 30% تا 70%  گزارش كرده اند. بر اساس مطالعات انجام شده وبه عقيده بيشتر نويسندگان، روش هاي قديمي براي كاهش علائم MPS  موقتي، ناقص و غير قابل اعتماد است؛ در صورتي كه استفاده از درمان هاي تداخلي  به عنوان درمان تكميلي و حتي درمان جايگزين نتايج مطلوبي در پي داشته است. بيشتر محققين اثرات درماني مثبت و يا منفي ليزر درماني را وابسته به روش و شدت انرژي تابيده شده مي دانند. بنابراين ضرورت انجام مطالعات بيشتر با روش ها و پارامترهاي  مختلف ليزر(شدت، طول موج، ديوريشن و ...) در بيماران مبتلا به MPS احساس مي شود. در اين مطالعه قصد داريم باانتخاب روش ليزر ‌آكوپانكچر، اثرات درماني ليزر كم توان Ga-Al-As را روي نقاط طب سوزني در بيماران مبتلا به سندرم درد مايوفاشيال در ناحيه گردن ، بررسي كنيم. مواد وروشها: اين مطالعه از نوع كارآزمايي باليني و به صورت يك سويه كور مي باشد. 60 بيمار زن مبتلا به MPS در رده سني 20 تا 60 سال  به طور تصادفي به دوگروه 30 نفري تقسيم شدند. در گروه اول از ليزر روشن و گروه دوم از ليزر خاموش روي نقاط طب سوزني به روش تماسي استفاده شد و هر دو گروه  تمرين كششي عضله تراپزيوس فوقاني را هر جلسه انجام مي دادند. درمان طي 10 جلسه  به صورت يك روز در ميان  توسط يك فيزيوتراپيست انجام مي شد. گردآوري اطلاعات مربوط به متغيرهاي وابسته(اندازه  گيري  شدت درد، دامنه حركتي فعال گردن و حساسيت نسبت به فشار بر روي نقاط ماشه اي) در 4 نوبت قبل از شروع درمان، جلسه 5، جلسه 10، و يك ماه پس از درمان با پرسشنامه توسط فيزيوتراپيست ديگري انجام مي شد. نتايج:استفاده از ليزر كم توان روي نقاط طب سوزني در بيماران مبتلا به سندرم درد مايوفاشيال در ناحيه گردن در كا هش درد حين فعاليت و استراحت ،افزايش دامنه حركتي فلكشن طرفي به چپ وراست و حساسيت نسبت به فشار در گروه درمان نسبت به گروه كنترل برتري نداشته است و هر دو گروه بهبودي نشان داده اند. ا ما در افزايش دامنه حركتي فلكشن ، اكستانسيون و چرخش به سمت راست وچپ موثر بوده است. بحث: هر چند بهبودي در كاهش درد، افزايش دامنه حركتي  وحساسيت نسبت به فشار در هر دوگروه ديده شد ولي  در گروه درمان نتايج بهتر از  گروه كنترل بود. علت بهبودي در هر دوگروه را مي توان به تاثير تمرين كششي و رو ش تماسي ليزر كه باعث تحريك نقاط طب سوزني مي شود نسبت داد.

Title: بررسي مدت زمان پاسخ دهي در تكليف ناميدن تصاوير اسامي در كودكان طبيعي

http://jrrs.mui.ac.ir/index.php/jrrs/article/view/138

چكيده زمينه و هدف: تکلیف نامیدن تصویر شیوه ای متداول جهت ارزیابی و درمان اختلالات زبانی در کودکان محسوب میشود. مدت زمان نامیدن تصویر در کودکان طبیعی معیاری مناسب جهت مقایسه ی این مهارت در کودکان دچار انواع اختلالات زبانی است. هدف از اين پژوهش، تعيين مدت زمان پاسخ دهي در تكليف ناميدن تصاوير اسامي در كودكان طبيعي 4 ساله و مقایسه ی این مقدار در دو جنس بود . روش بررسي: اين پژوهش ،يك مطالعه توصيفي و از نوع مقطعي مي باشد .در اين مطالعه 61 کودک 4ساله  مهدكودك هاي شهر اصفهان به صورت تصادفي انتخاب شدند . با استفاده ازنرم افزار DMDX براي هر كدام از نمونه ها مدت زمان پاسخ دهي به تصوير محاسبه شد .نمونه ها همه يك زبانه و سالم بودند. ابزار پژوهش 15 تصوير از تصاوير آشنا و دو هجايي بود. كودك بايد به محض نمايش تصوير بر روي صفحه كامپيوتر آن تصوير را مي ناميد. از نرم افزار SPSS   براي تجزيه و تحليل استفاده شد. يافته ها: ميانگين كل مدت زمان پاسخ دهي95/ 1682  محاسبه  شده است و  ميانگين مدت زمان پاسخدهي درپسران 5/1656و ميانگين مدت زمان پاسخ دهي در دختران4/ 1709 و56/ Pvalue= مي باشد كه نشان مي دهد كه ميانگين مدت زمان پاسخ دهي در دختر و پسر تفاوت معناداري ندارد . نتيجه گيري: كودكان 4 ساله طبيعي در ناميدن تصاوير كلمات دو هجايي به طور ميانگين سرعتي معادل 1682.95 دارندكه اين ميزان در دختران و پسران تقريبا" با هم برابر است.

Title: بررسی تاثیر آموزش تمرینات درمانی و مراقبت های عمومی از طریق پمفلت بر کاهش گردن درد

http://jrrs.mui.ac.ir/index.php/jrrs/article/view/139

مقدمه: یکی از مشاغلی که پتانسیل ایجاد مشکلات عضلاني-اسکلتی به ویژه گردن درد را دارد دندانپزشکی است.از آنجاییکه نتیجه قاطعی در مورد تأثیر مداخله آموزشی بر کاهش علائم گردن درد در مقایسه با سایر مداخلات درمانی وجود ندارد، هدف اصلی این مطالعه بررسی تأثیر آموزش تمرینات درمانی و مراقبت های عمومی گردن از طریق پمفلت بر میانگین مقیاس درد و ناتوانی گردن (NPDS) در دندانپزشکان می باشد. مواد و روش ها: این مطالعه از نوع تجربی آینده نگر و آسان می باشد. افراد مورد مطالعه را 30 دندانپزشک تشکیل می دادند که 23 نفر آنها مرد و 7 نفر آنها زن بودند.افراد مورد مطالعه به روش آسان از بین دندانپزشکان شهر اصفهان ضمن مراجعه به محل کار آنان در صورت دارا بودن شرایط ورود به مطالعه انتخاب شدند.پارامترهاي مورد بررسی در این تحقیق، میزان درد و ناتوانی ناشی از گردن درد بود که بوسیله ی پرسشنامه مقیاس درد و ناتوانی گردن (NPDS)  ارزیابی شد. جمع آوری اطلاعات و ارائه پمفلت آموزشی درمانی در طی سه مرحله قبل و بعد از مداخله آموزشی درمانی و 6 هفته پس از آن انجام شد. نتایج: نتایج این تحقیق نشان داد که آموزش از طریق پمفلت در دندانپزشکان مورد مطالعه با در نظر گرفتن دوره ی 4 هفته ای برای انجام تمرینات، نمره ی درد و ناتوانی گردن را در آنان بعد از مداخله ی آموزشی درمانی و همچنین در Follow up شش هفته ای نسبت به زمان قبل از مداخله ی به طور چشمگیری کاهش می دهد .(P-Value < 0/001) بحث و نتیجه گیری: توصیه می گردد به نقش آموزش بهای بیشتری داده شود.البته با توجه به افت تأثیر آموزش های پمفلت در زمان Follow up شش هفته ای نسبت به پایان دوره ی 4 هفته ای انجام تمرینات، پیشنهاد می شود که تمرینات پمفلت در مدت طولانی تری انجام شود و هر چند ماه یکبار تکرار تمرینات جهت یادآوری ضروری به نظر می رسد.

Title: بررسي ساختار سازه ای واکه های زبان فارسی در دانش آموزان مبتلا به شکاف لب و کام در محدوده ی سنی 9-7 ساله

http://jrrs.mui.ac.ir/index.php/jrrs/article/view/140

چكيده مقدمه:اهميت فركانس سازه اي به گونه اي است كه روابط ميان سه سازه اول از اجزاي اصلي تشخيص نوع واكه توسط فرد شنونده مي باشد. به علاوه كيفيت يك واكه به بسامد سازه هاي آن بستگي دارد، بنابراين كاربرد اصلي اندازه گيري بسامد سازه در توصيف خطاهاي واكه اي مي باشد. تشخيص غير طبيعي بودن واكه هاي يك فرد بر پايه هاي ادراكي توسط فرد شنونده نمي باشد ، بلكه اندازه گيري اين فركانس به كمك دستگاه تشخيص را دقيق تر مي كند. هدف از انجام این پژوهش به دست آوردن شاخص یا هنجارهایی جهت تشخیص خطاهای واکه ای و اختلالات گفتاری در دانش آموزان مبتلا به شكاف كام و لب است . روش پژوهش: این پژوهش توصيفي و به صورت مقطعی روي 23 نفر دانش آموز مبتلا به شكاف كام و لب در محدوده سني 9-7 ساله انجام گرفت.در اين پژوهش اطلاعات اوليه توسط مصاحبه مستقيم گرد آوري شده و سپس آزمون توليد  واكه هاي زبان فارسي از نمونه هاي مورد نظر بعمل آمد. سپس سازه اول و سازه دوم و سازه سوم هر شش واكه بطور جداگانه  توسط دستگاه speech studio و برنامه  real analysisنرم افزار Dr.speech   بدست آمد و اعداد مربوط به طور جداگانه در جدول مربوطه ثبت گرديد و با استفاده از روش هاي آماري توصيفي ( ميانگين ، انحراف معيار ، حداقل و حداكثر ) بر اساس اهداف پژوهش مورد تجزیه و تحلیل قرارگرفت. يافته ها: در دانش آموزان شكاف لب و كام ميانگين بيشترين مقدار سازه اول مربوط به واكه /æ/  685 هرتز ، سازه دوم مربوط به واكه /i/1542 هرتز و سازه سوم مربوط به واكه /i/ 3264 هرتز و ميانگين كمترين مقدار به ترتيب مربوط به واكه هاي /i/ 422 هرتز ، /u/  953 هرتز و /æ/ 2565 هرتز به دست آمد. بحث و نتيجه گيري : نتايج پژوهش حاضر نشان مي دهد كه ميزان سازه اول در واكه هاي /e/ ، /i/ افزايش داشته است ، پس احتمالا افراشتگي و ارتفاع زبان كاهش يافته است و ميزان  سازه دوم در واكه هاي /e/ ، /i/  كاهش چشمگيري داشته است ، در نتيجه زبان در واكه هاي پيشين به سمت عقب پسروي داشته است . البته با افزايش وسعت شكاف و افت شنوايي اين پسروي بيشتر شده است.

Title: بررسی پاسخ های بیانی کودکان 2/5 ساله ی فارسی زبان به آزمون تصویری بیان اسامی

http://jrrs.mui.ac.ir/index.php/jrrs/article/view/141

چکیده مقدمه: این پژوهش به منظور بررسی توصیفی پاسخ­های کود کان فارسی­زبان طبیعی 2/5ساله به آزمون تصویری بیان اسامی در حجم نمونه­ای مناسب از لحاظ آماری، انجام شد.  مواد و روش­ها: تعداد 60 کودک2/5 ساله از 32 مهد کودک در شهر اصفهان به روش نمونه­گیری خوشه­ای چندمرحله‌ای انتخاب شده و مورد ارزیابی قرار گرفتند. داده­های به دست آمده از آزمون تصویری بیان اسامی، با استفاده از روش­های آماری توصیفی (میانگین، انحراف معیار، حداقل و حداکثر نمرات، درصد پاسخ­های صحیح) مورد تجزیه و تحلیل قرارگرفتند. یافته­ها: این پژوهش نشان داد که آیتم­های مربوط به مقوله­ی «اعضای بدن» و «پوشاک»، از بیشترین درصد پاسخ­های صحیح در این گروه سنی برخوردارند. از طرفی آیتم­های انتزاعی، آیتم­هایی که اهمیت یا کاربرد کمی برایکودک در این سنین دارند و آیتم­هایی که کودک تجربه­ی اندکی از آنها دارد، همگی دارای درصدهای پایینی هستند. بحث و نتیجه­گیری: آزمون بیان اسامی به خوبی نمی‌تواند توانایی بیان اسامی را در گروه­ سنّي مورد مطالعه نشان دهد و دارای نواقصی چون کمی تعداد آیتم­ها در اکثر مقولات و کیفیت نامطلوب بعضی از تصاویر است که باید اصلاح شود. همچنین با توجه به داده­های این پژوهش می­توان به ویژگی­های اساسی ایتم­های انتخابی جهت آموزش اسامی پی برد.

Title: بررسی تأثیر اسپیلنت حمایت کننده مچ دست بر قدرت مشت كردن در بيماران مبتلا به سندروم کانال کارپ ایدیوپاتیک

http://jrrs.mui.ac.ir/index.php/jrrs/article/view/143

چکیده مقدمه: سندروم کانال کارپ از جمله ضايعاتي است كه شيوع نسبتا زيادي بخصوص در میانسالی و اغلب در خانمها دارد.  با توجه به اهميت مچ دست و نقش مهم آن در انجام فعاليت هاي روزانه و شيوع نسبتا بالاي اين عارضه در جامعه و بدنبال تحقيقات و درمانهاي غيرجراحي این عارضه بر آن شديم تا تأثير استفاده از اسپیلنت حمایت کننده مچ دست را بر کاهش درد ، كاهش علائم سندروم كانال كارپ و افزایش قدرت مشت كردن دست پس از استفاده از حمایت کننده های مچ دست در افراد مبتلا به سندروم کانال کارپ ایدیوپاتیک و كاهش علائم باليني و كلينيكي در اين ناحیه را بررسي كنيم. هدف: بررسی میزان تأثیر اسپیلنت حمایت کننده مچ دست برقدرت مشت كردن دربيماران مبتلا به سندروم کانال کارپ ایدیوپاتیک مواد و روشها:  28 بیمار مبتلا به سندروم کانال کارپ پس از گذراندن مراحل غربالگري، بطور تصادفی به دو گروه 14 نفره تقسیم شدند، به اینصورت که گروه اول از درمان اسپیلنت و گروه دوم بدون ورود مداخله کننده ها وارد تحقیق شدند و در قبل و 3 هفته پس از اعمال مداخله، اندازه قدرت مشت كردن دست، میزان شدت درد و بی حسی آنها مورد بررسي قرار گرفت. اسپیلنت ها مچ دست را در حالت نوترال قرار داده و بیماران این اسپیلنت ها را در طول شب استفاده کردند. نتایج:  میزان علائم بیماری (سوزش و مورمور شدن) بطور معنی داری در گروه آزمون کاهش یافت و قدرت مشت کردن افزایش یافت و بنابراین مشخص شد استفاده از اسپیلنت حمایت کننده مچ دست باعث کاهش علائم بیماری سندروم کانال کارپ می‌شود. بحث: بر اساس يافته ها استفاده از اسپيلنت حمايت كننده مچ دست مي‌تواند باعث كاهش علائم بيماري و افزايش قدرت مشت كردن و افزايش كارائي مچ دست گردد.

Title: بررسي ارتباط بين افسردگي و كيفيت زندگي در بيماران مبتلا به سكته مغزي

http://jrrs.mui.ac.ir/index.php/jrrs/article/view/144

مقدمه: سكته مغزي از علل عمده بروز معلوليت بوده ودر خيلي از موارد منجر به افسردگي پس از سكته مي‌گردد كه اثرات منفي شديدي را بر كيفيت زندگي مبتلايان به‌همراه دارد. افسردگي پس از سكته و كيفيت زندگي ممكن است تاثيراتي متقابل بر هم داشته باشند (هركدام باعث تشديد ديگري و بدتر شدن وضع بيمار شود). با توجه به اينكه تاكنون تحقيقات زيادي در اين خصوص صورت نگرفته است، لذا لازم است كه ارتباط بين افسردگي و كيفيت زندگي در اين بيماران مورد ارزيابي قرار گيرد مواد و روشها: در مطالعه اي مقطعي، ارتباط بين افسردگي پس از سكته و كيفيت زندگي در بيماراني كه در بيش از شش ماه از سكته آنها ميگذشت، بررسي گرديد. آزمودني ها از بيماران مبتلا به سكته مغزي مراجعه كننده به دو مركز توانبخشي بيماران سرپايي در تهران، مركز رفيده و مركز تبسم بودند. براي ارزيابي كيفيت زندگي از پرسشنامه كيفيت زندگي SF-36 و براي بررسي خلق بيماران از پرسشنامه بك (BDI)  استفاده شد. در مجموع 40 بيمار مبتلا به سكته مغزي (21مرد و 19 زن) با ميانگين سني 3.5±61.5 سال  كه بطور ميانگين 3.5±8.7 ماه از سكته آنها ميگذشت ارزيابي شدند نتايج: در حدود 70.4% افراد داراي افسردگي بودند. بيماران افسرده بطور معناداري در تمامي معيارهاي تست SF-36 داراي نمره پايين تر بودند. يك همبستگي معكوس و منفي قوي بين افسردگي پس از سكته و كيفيت زندگي وجود داشت بطوريكه در تمامي خرده آزمونهاي كيفيت زندگي ضرايب همبستگي در سطح P<0.001معنادار بودند بحث: بخش قابل ملاحظه اي از بيماران مبتلا به سكته مغزي مراجعه كننده به كلينيكهاي توانبخشي دچارافسردگي پس از سكته هستند كه اين افسردگي تاثيري منفي و شديد بر كيفيت زندگي آنها دارد. بنابراين لازم است كه در آينده تحقيقات بيشتري بر روي افسردگي صورت گرفته تا رابطه آن با كيفيت زندگي مشخص تر گردد

Title: ارتباط بین مهارت خواندن با حافظۀ بینائی ناکلمه ها در دختران پایه اول ابتدايي

http://jrrs.mui.ac.ir/index.php/jrrs/article/view/146

چكيده: خواندن یکی از اصلی ترین ابزارهای یادگیری می باشد.همچنین از مهم ترین مهارتهایی که دانش آموزان در دورۀ ابتدائی به آن دست می یابند ، یادگیری خواندن است. شکست تحصیلی تا حد زیادی به سبب بی کفایتی مهارتهای خواندن رخ می دهد. از سوی دیگر در همۀ یادگیریها حافظه نقش مهمی را ایفا می کند و مشکلات حافظه در بسیاری از کودکان مبتلا به ناتوانیهای یادگیری با فرایندهای دیداری یا شنیداری گوناگون پیوند دارد و نیز امکان دارد دانش آموزان مبتلا به کاستیهای حافظه دیداریاز تشخیص حروف در کلمات و یا توالی صداها در یک کلمه با مشکل روبرو  شوند. مواد و روش‌ها: این مطالعه از نوع مقطعی و توصیفی – تحلیلی بوده است. تعداد 100نفراز  دانش آموزان دختر پایه اول ، شهر اصفهان به طور تصادفي انتخاب شدند كه براي اولين بار  در پايه اول درس مي خواندندو از نظر بینائی و شنیداری وبهره هوشي سالم بوده و مشکلات خواندن و اختلالات تولیدی یا روانی گفتار نداشتند.آزمون خواندن در مورد هر نمونه اجرا و  در حين خواندن صدای او ضبط می شد وسپس باشمارش  تعداد خطاها نمره درستی خواندن وبا گرفتن زمان خواندن متن نمره سرعت خواندن محاسبه گرديد. آزمون حافظه بينايي ناكلمه ها اجراونتايج آن ثبت گرديد.  يافته ها: نشان داد بین درستی خواندن و حافظه بینائی ناکلمه ها در نمونه های مورد بررسی ارتباط معنی داری وجود دارد(318 r=/ و01/0p=). بین نمره سرعت خواندن و نمره حافظه بینائی ناکلمه ها در نمونه های مورد بررسی همبستگی وجود ندارد(051r=/ و61/0p=). نتيجه گيري : در تحليل بدست آمده از اين پژوهش مي توان گفت مهارتهایی نظیر رمز گشایی و حافظه بینائی هر دو در امر خواندن ، بخصوص ، در درستی خواندن نقش مهمی را دارند
